# Increased brain cytokine level associated impairment of vigilance and memory in aged rats can be alleviated by alpha7 nicotinic acetylcholine receptor agonist treatment

**DOI:** 10.1007/s11357-023-01019-6

**Published:** 2023-11-23

**Authors:** Zsolt Kristóf Bali, Lili Veronika Nagy, Nóra Bruszt, Kornélia Bodó, Péter Engelmann, Zsófia Hernádi, Kitti Göntér, Sai Ambika Tadepalli, István Hernádi

**Affiliations:** 1 Grastyán Endre Translational Research Centre, University of Pécs, Pécs, Hungary; 2 Translational Neuroscience Research Group, Centre for Neuroscience, Szentágothai Research Centre, University of Pécs, Pécs, Hungary; 3 Department of Neurobiology, Faculty of Sciences, University of Pécs, Pécs, Hungary; 4 Institute of Physiology, Medical School, University of Pécs, Pécs, Hungary; 5 Department of Immunology and Biotechnology, Medical School, University of Pécs, Pécs, Hungary; 6 Present Address: National Laboratory of Virology, Szentágothai Research Centre, University of Pécs, Pécs, Hungary

**Keywords:** Aging, Neuroinflammation, Alpha7 nicotinic acetylcholine receptor, Vigilance, Memory, Neurocognitive disorders

## Abstract

Age-related neurocognitive disorders are common problems in developed societies. Aging not only affects memory processes, but may also disturb attention, vigilance, and other executive functions. In the present study, we aimed to investigate age-related cognitive deficits in rats and associated molecular alterations in the brain. We also aimed to test the effects of the alpha7 nicotinic acetylcholine receptor (nAChR) agonist PHA-543613 on memory as well as on the sustained attention and vigilance of aged rats. Short- and long-term spatial memories of the rats were tested using the Morris water maze (MWM) task. To measure attention and vigilance, we designed a rat version of the psychomotor vigilance task (PVT) that is frequently used in human clinical examinations. At the end of the behavioral experiments, mRNA and protein expression of alpha7 nAChRs, cytokines, and brain-derived neurotrophic factor (BDNF) were quantitatively measured in the hippocampus, frontal cortex, striatum, and cerebellum. Aged rats showed marked cognitive deficits in both the MWM and the PVT. The deficit was accompanied by increased IL-1beta and TNFalpha mRNA expression and decreased BDNF protein expression in the hippocampus. PHA-543613 significantly improved the reaction time of aged rats in the PVT, especially for unexpectedly appearing stimuli, while only slightly (non-significantly) alleviating spatial memory deficits in the MWM. These results indicate that targeting alpha7 nAChRs may be an effective strategy for the amelioration of attention and vigilance deficits in age-related neurocognitive disorders.

**Supplementary Information:**

The online version contains supplementary material available at 10.1007/s11357-023-01019-6.

## Introduction

Aging is a complex and multifactorial process induced by the multifaceted interaction of genetic, epigenetic, and environmental factors, and it appears as a consequence of the action of time on living organisms [1]. During aging, the functional capabilities of the brain decline progressively, which generally affects cognitive performance, including attention, speed of information processing, short-term (working) memory, long-term memory, and executive functions [2].

Advancing age is the strongest known risk factor for most neurodegenerative disorders, including disorders of the mind termed neurocognitive disorders (NCDs), as the prevalence of these conditions increases significantly with age even without other identifiable risks [3]. Despite its central role in the pathogenesis of NCDs, the role of aging of the brain has not been fully understood at cellular and molecular levels [4]. Therefore, to develop successful diagnostic tools and therapeutic interventions, it is important to consider the basic cross-species hallmarks of aging at the cellular and molecular levels in various tissues and their causal role in the onset and progression of NCDs in humans, as well as in preclinical (translational) model organisms [5].

Similar to the hallmarks of aging in other tissues, the aging brain also shows mitochondrial dysfunction, dysregulated energy metabolism, compromised DNA repair, aberrant neuronal network activity, dysregulated Ca^2+^ handling, and inflammation [6]. The activation of microglia and astrocytes is one of the fundamental events in the progressive neuroinflammation associated with aging, often termed inflammaging [7]. Microglia readily transform into an activated state in the aged brain, which is characterized by their amoeboid morphology in response to neuronal injuries or neurodegeneration and by the excessive production of proinflammatory cytokines, including interleukin-1beta (IL-1beta), IL-6, and tumor necrosis factor alpha (TNFalpha), which have detrimental effects on brain health, particularly during the aging process. [6, 8].

The aging of the brain is also generally associated with increased neuronal vulnerability, which differentially affects the different brain areas and associated neurotransmitter systems. For instance, the cholinergic system becomes particularly vulnerable during aging and undergoes moderate to severe degenerative remodeling in the basal forebrain, resulting in marked cell loss and hypofunction [9, 10]. There is a clear relationship between cholinergic hypofunction and a decline in cognitive functions, especially attention, learning, and memory. Furthermore, the administration of cholinergic agonists to animals and elderly human subjects has been suggested to result in a moderate improvement in various cognitive functions [11, 12]. Alpha7 nicotinic acetylcholine receptors (nAChR) are highly expressed in the nervous system, mostly in regions involved in cognition and memory processes (prefrontal cortex, hippocampus) and their dysfunction has been associated with several neurocognitive and neuropsychiatric disorders [13–15]. Furthermore, besides being abundant in neurons of memory-relevant cortical areas, alpha7 nAChRs are also expressed in microglia and astrocytes and significantly affect their polarization, antigen presentation, and cytokine production [16]. Therefore, alpha7 nAChR-specific agents have been brought into the focus of drug development for the treatment of NCDs both in preclinical model organisms and in clinical trials [17, 18].

In line with the above, the current study aimed to investigate both behavioral and molecular-level (mRNA and protein expression) alterations in the aged rat brain and to assess the effectiveness of an alpha7 nAChR-targeting cognitive enhancer treatment to predict the beneficial role of pharmacological alpha7 nAChR-modulation in pathological aging. Therefore, we first determined the cognitive deficits of aged rats using the Morris water maze (MWM) and the psychomotor vigilance task (PVT). Then, we investigated the effects of an alpha7 nAChR orthosteric agonist on spatial memory and vigilance in the aged rats. After the behavioral pharmacological studies, we assessed the mRNA and protein expression of IL-1beta, IL-6, TNFalpha, BDNF, NFkappaB, and alpha7 nAChR in key areas of the brain.

## Materials and Methods

### Animals

Male Lister hooded rats (EnVigo, Horst, Netherlands) were used in the present study. Aged animals were courtesy of Gedeon Richter Plc., where they participated in behavioral pharmacological experiments. They spent at least 6 months accommodating to our animal house before the experiments started. Morris water maze experiments were started with 36 naturally aged (25 months old) and 20 young adult (6 months old) rats. Twelve aged and twelve young rats were trained for the PVT. The PVT training started when aged rats were 31 months old and young adult rats were 12 months old, and the training lasted 2 months. Thus, aged rats were 33 months old, and young adult rats were 14 months old when the PVT experiments comparing the performance of aged and young rats were conducted. Then, the number of aged animals rapidly decreased between the ages of 33 and 34 months because of natural death. At the end of the experiments, aged rats were 34 months old, while young adult rats were 15 months old when 4 aged and 4 young adult rats were sacrificed for the analysis of brain samples using PCR and ELISA assays.

During the entire study, the animals were pair-housed under a 12/12 h daily light/dark cycle with controlled temperature and humidity in the animal house of the Szentágothai Research Centre, University of Pécs, Hungary. In the animal house, lights were ON from 7 a.m. to 7 p.m., and the animals were tested during the light ON period. Rats were fed daily with a controlled amount of food (17 g/animal laboratory chow, Ssniff Rat/Mouse Maintenance chow, Ssniff Spezialdiäten GmbH, Soest, Germany) to prevent the development of obesity and other related health problems. Water was available ad libitum. The experiments were approved by the Animal Welfare Committee of the University of Pécs and the National Scientific Ethical Committee on Animal Experimentation (ÁTET) of the Ministry of Agriculture (ethical license no.: BA02/2000–25/2015). All procedures fully complied with Decree No. 40/2013. (II. 14.) of the Hungarian Government and the EU Directive 2010/63/EU on the Protection of animals used for scientific purposes.

### Morris water maze task

First, the spatial memory performance of aged and young rats was compared using the Morris water maze task (MWM). After confirming the memory deficit of aged rats in the first MWM experiment compared to young rats, a new experiment was designed to test the effects of the alpha7 nAChR agonist PHA-543613 on the memory performance of the same aged rats in the MWM.

The protocol of the MWM task was the same as described in previous reports from our laboratory [19, 20]. The task is represented in Supplementary Fig. 1A. In brief, experiments were carried out in a blue circular pool, 180 cm in diameter and 90 cm in height (Ugo Basile, Gemonio, Italy), filled to a depth of 30 cm with blue-painted, opaque water containing 500 g milk powder and 30 ml blue food coloring. The area of the pool was divided into four virtual quadrants (NW, SW, SE, NE). The experiments were recorded using Ethovision XT10 software (Noldus, Wageningen, Netherlands).

The experimental protocol started with daily training sessions for four consecutive days (with one 4-trial session each day), when a platform was placed in the center of one of the quadrants (submerged 1 cm below the surface), and the rats had to learn the location of the hidden platform with the help of visual cues placed around the maze. In the first MWM experiment (comparing aged and young rats), the platform was placed in the SW quadrant. In each trial, the rats were placed in different quadrants changed clockwise from trial to trial (starting points: N, E, S, W), and were allowed to search for the hidden platform for 2 min. During the trials, we measured the escape latency, which is the time elapsed until the animal found the platform. Furthermore, the swimming path length was also analyzed on training days using Ethovision XT10 software. If the platform was not found during the trial, the rat was placed on the platform for 10 s, and 2 min was recorded as escape latency. The performance of the animals on the first training day was used to evaluate their short-term memory performance, and changes in their average daily performance represented the learning process. On the fifth day, a single probe trial was performed to test the recall of long-term memory, when the platform was removed from the pool and the animals were allowed to search the pool for 2 min. During the probe trial, the time spent in the target quadrant (the quadrant where the platform was located during the training sessions) was measured as an index of long-term memory recall. Before the second MWM experiment testing the effects of PHA-543613, three more MWM sessions were run for the purpose of extinction of the memory trace for the earlier location of the platform. The extinction trials were run in the same manner as the probe trial.

Next, the effects of PHA-543613 were tested in the MWM using a between-subject design. Aged animals were treated on each of the four consecutive training days with one of the following treatments 45 min prior to testing: saline (A_VEH), 1.0 mg/kg PHA-543613 (A_PHA1), 3.0 mg/kg PHA-543613 (A_PHA3). Young control rats were treated with saline only (Y_VEH). The solutions were applied at 1 ml/kg dosing volume. The effects of PHA-543613 were evaluated in four training days (when the platform was hidden in the NE quadrant) and in a probe trial conducted on the fifth consecutive day (when the platform was absent).

### Psychomotor vigilance task

The psychomotor vigilance task (PVT) was conducted as described in a recent study from our laboratory [21]. The apparatus and the task protocol are represented in Supplementary Fig. 1B and C, respectively. In brief, for conditioning, we used a standardized operant conditioning apparatus for rats (Habitest System, Coulbourn Instruments, Holliston, MA), equipped with stimulus generators (feeder light, color LED lights), response sensors (levers, photocell) and reward pellet delivery modules (feeder with delivery trough). Twelve aged and twelve young rats were gradually trained in a 10-step procedure (detailed in the Supplementary Material) to fully perform the PVT. All of the animals were successfully trained for the PVT.

Every trial of a PVT session was run as follows: rats were required to respond to the onset of the centrally located feeder light module by nose-poking into the pellet delivery trough of the feeder module. Then, the rats had to hold their nose in the delivery trough until the feeder light was turned off (fixation). The fixation time (foreperiod) varied randomly between 0 and 5000 ms. At the end of the foreperiod, the feeder light turned off and the color LED lights were illuminated above the two levers located left and right to the central feeder module serving as a cue signal for the rats to pull their nose out of the pellet delivery trough and press one of the levers within 10 s. After the lever press response, rats immediately received a reward pellet (45 mg dustless precision pellet, Bio-Serv, Flemington, NJ), the color LED lights were also turned off, and an intertrial interval (ITI) of 15 s started. If the animal correctly accomplished the given trial, the total reaction time (RT) was measured as the time from the offset of the feeder light (onset of the cue lights) until the lever press response. In the analysis, RT was split into two parts: 1) the time from the cue (feeder light off, color LEDs on) until pulling the nose out of the pellet delivery trough was considered as a decision phase of the response and was referred to as “noseout” time; while 2) the time from pulling the nose out until pressing the lever was considered as a mainly motor part of the response and was referred to as “pressing speed”. If the trial was not correctly performed, 3 different kinds of errors were defined and registered. If the animal did not put its nose in the feeder trough within 10 s after the onset of the feeder light, the trial was not initiated, and it was considered a “missed trial”. If the animal nose-poked but removed its head from the feeder trough before the offset of the feeder light, a “premature response” was recorded. If the animal succeeded the foreperiod but did not press the lever after the onset of the cue lights, it was considered an “omission error or lapse”. All types of errors resulted in the offset of all light stimuli and a 5 s extra timeout (punishment) period followed by the normal ITI of 15 s. In each experimental session, rats performed the task for 60 min or until 90 initiated trials were reached.

The performance of aged and young rats was compared in 3 sessions on 3 different days, and the recorded data was averaged between testing days. At this part of the study, aged rats were 33 months old, and young rats were 14 months old. Then, the effects of alpha7 nAChR agonist PHA-543613 on the performance of aged rats were tested in a within-subject experimental design. The aged rats performed four PVT sessions and the following treatments were applied in a randomized counter-balanced latin-square design: saline (VEH), 0.3 mg/kg PHA-543613 (PHA0.3), 1.0 mg/kg PHA-543613 (PHA1.0), 3.0 mg/kg PHA-543613 (PHA3.0).

### Brain tissue collection and preparation

After completing the behavioral experiments, animals were anesthetized with an overdose of pentobarbital (800 mg/kg, i.p.) and were transcardially perfused. Four young and four aged rats were perfused with 0.9% saline, their brains were rapidly removed and were bilaterally dissected to the following brain areas: cerebellum (CER), hippocampus (HC), striatum (STR), and neocortex (CTX, frontal lobe). The dissected brain samples were frozen immediately in liquid nitrogen and were stored at -80 °C until biochemical analyses were performed.

### Protein isolation and ELISA test

Total protein from the different brain regions was isolated in RIPA lysis buffer (50 mM Tris–HCl, pH: 8.0, 150 mM NaCl, 1% NP-40, 0.5% Na-deoxycholate, 5 mM EDTA, 0.1% SDS) with the addition of protease inhibitor cocktail (Sigma-Aldrich). The total protein concentrations of the samples were measured using a BCA Protein Assay kit (Pierce, Rockford, IL). Quantification of IL-1beta, BDNF, and alpha7 nAChR was carried out using Abbexa (Cambridge, UK) ELISA kits with the following catalog numbers, respectively: abx155713, abx155251, abx556026. ELISAs were performed according to the manufacturer’s protocols. Immediately after developing the final color reaction of the assay, the absorbance was measured at 450 nm with an iEMS Reader microplate reader (Thermo Fisher Scientific).

### RNA isolation and quantitative RT-PCR measurements

The total RNA of the brain samples was extracted according to the manufacturer’s protocol by applying the NucleoSpin RNA kit (Macherey–Nagel, Düren, Germany). The quality and quantity of RNA were assessed at 260 nm using a NanoDrop 1000 spectrophotometer (Thermo Fisher Scientific, Waltham, USA). The cDNA was constructed from total RNA with High Capacity cDNA reverse transcription kit (Thermo Fisher Scientific) in 20 μl reactions using random hexamers following the manufacturer’s protocol. The resulting cDNA was stored at − 20 °C. Target gene expressions were measured by real-time PCR using Maxima SYBRGreen MasterMix (Applied Biosystems, Waltham, USA) with an ABI Prism 7500 instrument (Applied Biosystems). The cDNAs were applied as a template for the amplification reactions. All samples were tested in duplicates. Primers were designed in Primer Express software (Thermo Fisher Scientific) considering the exon–intron boundaries for all target genes (Table 1). Cyclophilin A (CycA) was used as a housekeeping gene for the quantification of RNA. The thermal profile started at 95 ºC for 10 min, 40 cycles of 35 s at 95 ºC, 35 s at 60 ºC, and 1 min at 72 ºC.

**Table 1:** Primer sequences applied for qPCR

Target gene	GenBank Acc#	Primers (5´-3´)^*a*^	Amplicon size (bp)
Cyclophilin A	BC059141	GGA AGC CAT GGA GCG TTT T AAT GCC CGC AAG TCA AAG AA	100
Nicotinic receptor α7	S53987	AGT GCT GCA AAG AGC CAT ACC ATG AGT ACA CAA GGG ATG AGC AGA T	100
BDNF	M61175	CAC TTT TGA GCA CGT GAT CGA CAC CCG GGA AGT GTA CAA GTC	100
NF-kB	NM_199267	ACC TGG AGC AAG CCA TTA GC CGG ACC GCA TTC AAG TCA TA	100
IL-1 beta	NM_031512	GAG TCT GCA CAG TTC CCC AA ATG TCC CGA CCA TTG CTG TT	100
IL-6	NM_012589	CAC AAG TCC GGA GAG GAG AC GCC ATT GCA CAA CTC TTT TCT CA	133
TNF-alpha	NM_012675	CAG CAA CTC CAG AAC ACC CT GGA GGG AGA TGT GTT GCC TC	104

^*a*^Upper and lower sequences represent forward and reverse primers, respectively

### Data analysis and statistics

Data analysis and statistical tests were made in Microsoft Excel and Jamovi 2.2.5 [22].

In the MWM, training day performance was expressed as the time needed to find the platform (escape latency) and was analyzed using repeated-measures ANOVA with TRIALS or DAYS as a within-subject factor and AGE as a between-subject factor. Performance in probe trials (including extinction training) was expressed as the time spent in the target quadrant (where the platform was earlier located) and was analyzed using independent samples t-test or one-way ANOVA following post-hoc LSD tests.

In the PVT, total reaction time and its components as well as the number of premature errors were averaged over all trials or trials with a given range of foreperiod length. In the latter case, performance was analyzed as a function of foreperiod length using a linear mixed-effects model. Data averaged over all trials were analyzed using independent samples t-test (aged vs. young) or linear mixed-effects model (effects of different doses of PHA-543613) with animal identity as a random intercept. The omnibus test of the linear mixed-effects model was followed by LSD post-hoc comparison.

Protein concentrations measured in the brains of aged and young rats were compared using independent samples t-test. Quantitative PCR data were expressed as arbitrary units that indicate the mRNA expression of target genes normalized to CycA housekeeping gene and were analyzed using independent samples t-test.

After omnibus tests (i.e., ANOVA and linear mixed-effects model) pairwise comparisons were made between the levels when the main effect showed at least a tendency (*p* < 0.1) to a significant effect. Pairwise comparisons were considered significant if *p* < 0.05.

## Results

### Morris Water Maze test

#### Spatial memory performance of aged and young adult rats

**Fig. 1:**
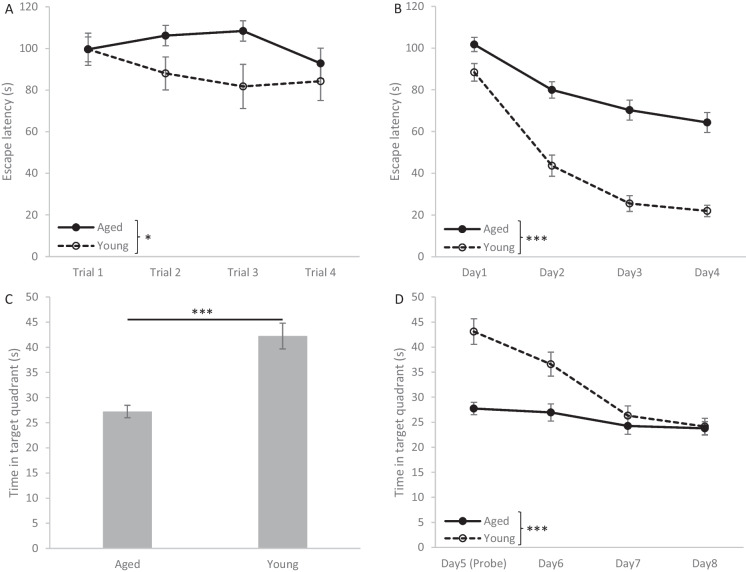
Performance of aged and young rats in the Morris water maze (MWM). **A**: Time needed to find the platform (escape latency) in the four trials of the first training day. **B**: Average escape latency on the four days of the training process. **C**: Performance in the probe trial (5th day) when the platform was not placed in the pool. **D**: Extinction of memory for platform location in four consecutive probe trials (platform is absent). Asterisks mark significant differences between aged and young rats: * *p* < 0.05, *** *p* < 0.001

In the first MWM experiment, the memory performance of aged and young adult rats was compared (Fig. 1). One aged rat was excluded from the study because it could not swim. On the first training day, aged rats performed overall worse than young rats as they needed significantly more time to find the hidden platform (AGE: F(1, 53) = 4.967, *p* = 0.030; Fig. 1A). The difference between the performance of aged and young rats further increased during the consecutive training days (Fig. 1B). Aged rats showed less improvement during training days than young rats as the learning curve of young rats was significantly steeper than that of aged rats (DAY × AGE: F(3, 159) = 7.571, p < 0.001). The overall performance of aged rats on the four training days was also significantly worse compared to young rats (AGE: F(1, 53) = 71.004; p < 0.001). Furthermore, aged rats also showed significant long-term memory deficits in the probe trial (Fig. 1C), when they searched the platform in the target quadrant for a shorter time than young rats (t(53) =  − 5.991, p < 0.001). The results of the extinction training (Fig. 1D) also confirmed that aged rats poorly learned the location of the platform. During the four sessions, when the platform was absent, young rats spent gradually less time day by day searching the platform in its previous location, while the time spent in the target quadrant decreased in a significantly smaller extent in the case of aged rats (DAY × AGE: F(3, 156) = 11.570, p < 0.001). Eventually, both aged and young rats spent almost equal time in the target quadrant (indicating complete extinction of their searching behavior in the previous location of the platform), but in the case of aged rats the time spent in the target quadrant was only slightly less than in the probe trial, also indicating their impaired spatial learning. In summary, short-term memory, learning (acquisition), and long-term memory (recall) were all found impaired in aged rats in the MWM task.

#### Effects of PHA-543613 on the spatial memory of aged rats

**Fig. 2:**
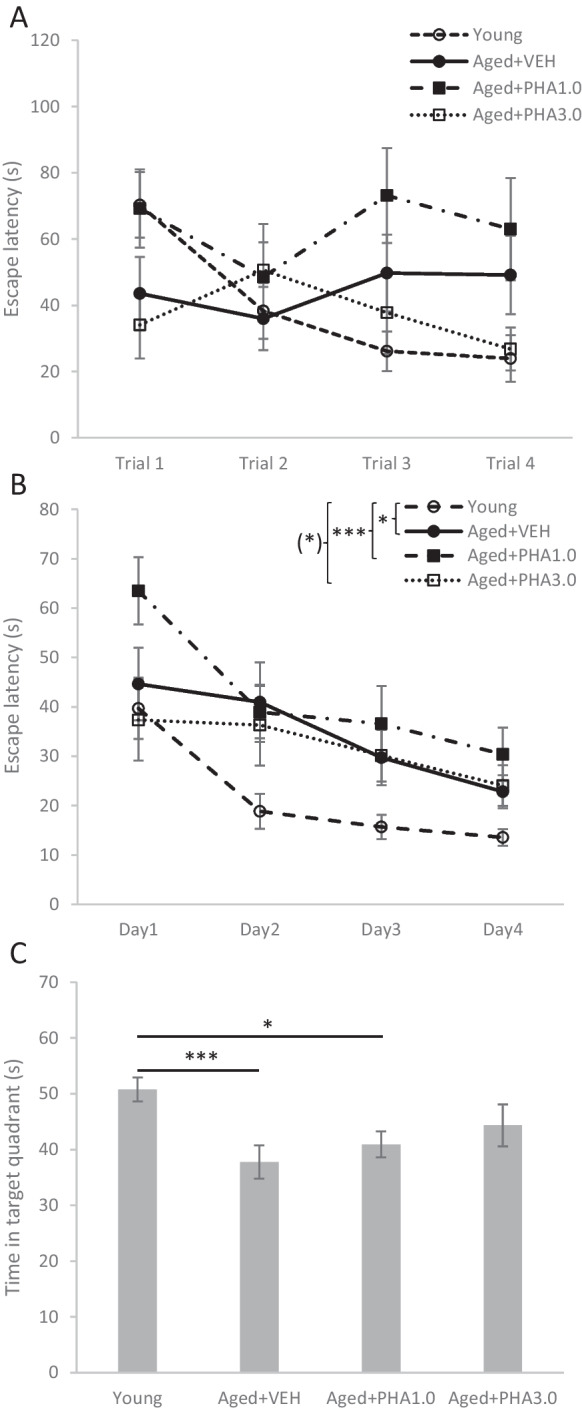
Effects of alpha7 nicotinic acetylcholine receptor agonist PHA-543613 on the memory performance of aged rats in the water maze. **A**: Time needed to find the platform (escape latency) in the four trials of the first training day. **B**: Average escape latency on the four days of the training process. **C**: Performance in the probe trial (5th day) when the platform was not placed in the pool. Asterisks mark significant differences between experimental groups: (*) *p* < 0.1, * *p* < 0.05, *** *p* < 0.001

Next, we tested the effects of alpha7 nAChR agonist PHA-543613 on the performance of aged rats in the MWM (Fig. 2). Results of the first training day (Fig. 2A) showed that the short-term memory performance of young adult rats was significantly better than that of other groups (TRIAL × AGE: F(9,150) = 2.600, *p* = 0.008). Only young rats improved from trial to trial on the first day (F(3, 54) = 13.760, p < 0.001), aged rats showed no improvement regardless of the applied treatments (A_VEH: F(3, 33) = 0.448, *p* = 0.720; A_PHA1: F(3, 33) = 0.691, *p* = 0.564; A_PHA3: F(3, 30) = 1.334, *p* = 0.282). Although all four groups showed improvement during the four consecutive training days (Fig. 2B), the overall performance (average escape latency) of the animals was different depending on their age and the applied treatments (GROUP: F(3, 50) = 5.536, *p* = 0.002). Young rats found the platform significantly faster than aged rats treated with vehicle or 1.0 mg/kg PHA-543613 (Y_VEH vs. A_VEH: *p* = 0.018, Y_VEH vs. A_PHA1: p < 0.001). However, no significant difference was found between the overall escape latency of young rats and aged rats treated with 3.0 mg/kg PHA-543613 (Y_VEH vs. A_PHA3: *p* = 0.065). On the other hand, no significant difference was found between the escape latency of vehicle-treated and PHA-543613-treated aged rats. On the fifth experimental day, the long-term spatial memory of the rats was tested in a probe trial, when the platform was absent (Fig. 2C). Young rats spent significantly more time seeking the platform in the target quadrant than aged rat groups treated with vehicle or 1.0 mg/kg PHA-543613 (F(3, 50) = 4.850, *p* = 0.005; Y_VEH vs. A_VEH: p < 0.001; Y_VEH vs. A_PHA1: *p* = 0.010). However, there was no significant difference between the performance of aged rats treated with 3.0 mg/kg PHA-543613 and young rats (Y_VEH vs. A_PHA3: *p* = 0.094). On the other hand, no significant difference was found in the time spent in the target quadrant between vehicle-treated and PHA-543613-treated aged rats. In the probe trial, the swimming speed of the rats was also compared, and we found that there was no difference between the swimming speed of young, vehicle-treated aged, and PHA-543613-treated aged rats indicating that PHA-543613 did not exert its effect through the general increase of locomotor speed of animals. In summary, different measurement endpoints of the MWM task initially showed marked cognitive (memory performance) deficit in aged rats which was only slightly alleviated by the high but not the low dose of PHA-543613.

### Psychomotor Vigilance Task

#### Comparison of PVT performance of aged and young adult rats

**Fig. 3:**
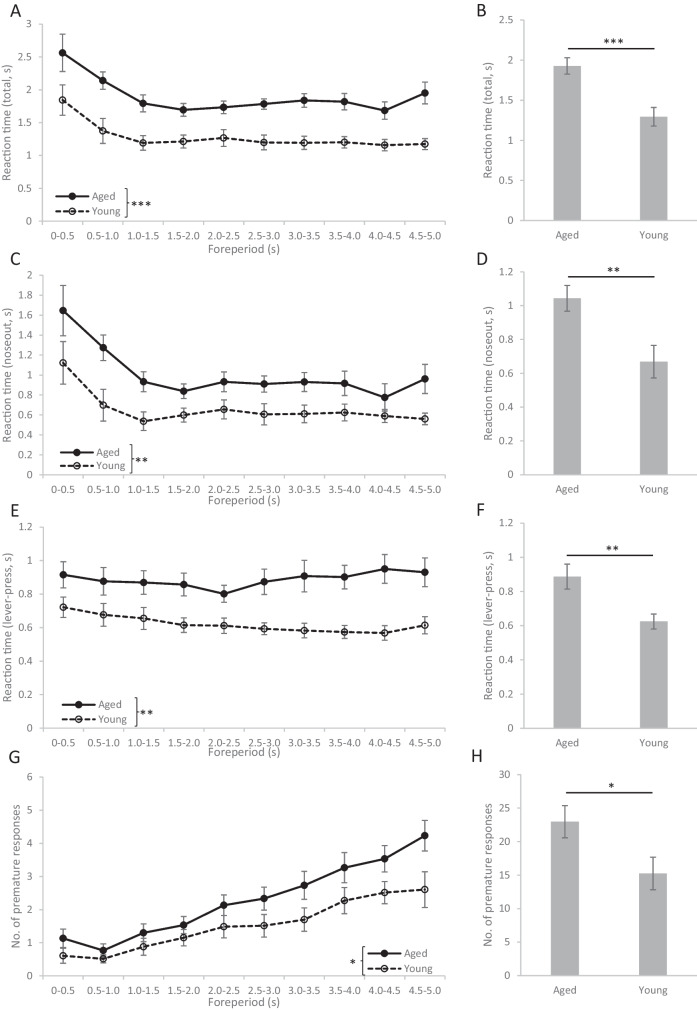
Performance of aged and young rats in the psychomotor vigilance task. **A**: Total reaction time (from the cue until pressing the lever) as a function of foreperiod length. **B**: Total reaction time averaged over all trials. **C**: Time elapsed from cue presentation until pulling the nose out of the feeder trough (noseout) as a function of foreperiod length. **D**: Noseout part of the reaction time averaged over all trials. **E**: Time elapsed from pulling the nose out of the feeder trough until pressing the lever (lever-pressing) as a function of foreperiod length. **F**: Lever-pressing part of the reaction time averaged over all trials. **G**: Number of premature responses as a function of foreperiod length. **H**: Total number of premature responses. Asterisks mark significant differences between aged and young rats: * *p* < 0.05, ** *p* < 0.01, *** *p* < 0.001

Out of the 12 aged rats that were trained for the PVT, two rats showed very low motivation and performed an insufficient number of correct trials. Therefore, these two animals were excluded from the analysis. Aged rats responded to the randomly timed stimuli significantly slower than young rats (t(19) = 4.082, p < 0.001, Fig. 3A-B). Analysis of the reaction time as a function of the foreperiod showed typical effects of expectation for stimuli appearing after a longer foreperiod compared to unexpectedly rapid stimuli (Fig. 3A). Namely, reaction time showed a gradual decrease between 0 to 1.5 s foreperiod (F(9, 168.1) = 12.145, p < 0.001; 0–0.5 vs. 0.5–1.0 s: p < 0.001; 0.5–1.0 vs. 1.0–1.5 s: *p* = 0.006). However, increasing the foreperiod over 1.5 s did not result in any further decrease in reaction time (1.0–1.5 vs. 1.5–2.0 s: *p* = 0.680) indicating that the maximal speed of rats for responding to the stimulus was already reached. The stimulus expectation (foreperiod) effect was detected both in aged and young rats as no interaction was found between the effects of foreperiod length and age (FOREPERIOD × AGE: F(9, 168.1) = 0.586, *p* = 0.807). Thus, age did not affect the ability of rats to respond faster to expected (late) stimuli and did not affect the length of foreperiod where the maximum speed of responses was reached. Similarly, the expectation effect was detected in both age groups when only the decision phase (“noseout” response) of the reaction time was analyzed (FOREPERIOD: F(9, 168.1) = 10.701, p < 0.001; FOREPERIOD × AGE: F(9, 168.1) = 0.917, *p* = 0.512; Fig. 3C). Analysis of the “noseout” time confirmed the deficit of aged rats to respond to the stimuli as the decision phase of the reaction time was also significantly slower than in young rats (t(19) = 3.004, *p* = 0.007; Fig. 3D). However, the motor phase of the responses (“lever-pressing”) was differently influenced by the foreperiod in aged and young rats (FOREPERIOD × AGE: F(9, 168) = 2.483, *p* = 0.011; Fig. 3E). The lever-pressing time of young rats slightly decreased for stimuli preceded by long foreperiods indicating the development of an expectation of the forthcoming cue signal (F(9, 90) = 3.076, *p* = 0.003). Such expectation effect was completely absent in the motor phase of responses of aged rats (F(9, 78) = 1.705, *p* = 0.102). Furthermore, aged rats pressed the lever significantly slower than young rats (F(1, 19) = 11.060, *p* = 0.004), thus, aged rats exhibited a marked decline in the speed of motor execution, too.

Slower responses of aged rats were also accompanied by more premature errors (t(19) = 2.260, *p* = 0.036; Fig. 3H) indicating that aged rats badly tolerated long waiting times for the appearance of the cue stimuli. Furthermore, aged rats initiated fewer trials than young rats, possibly indicating their declined motivation and/or increased mental fatigue (t(19) = 3.186, *p* = 0.005, Fig. 5A). The number of omission errors was not statistically evaluated since both aged and young rats made very few or (mostly) no omission errors.

#### Effects of PHA-543613 on the performance of aged rats in the PVT

**Fig. 4:**
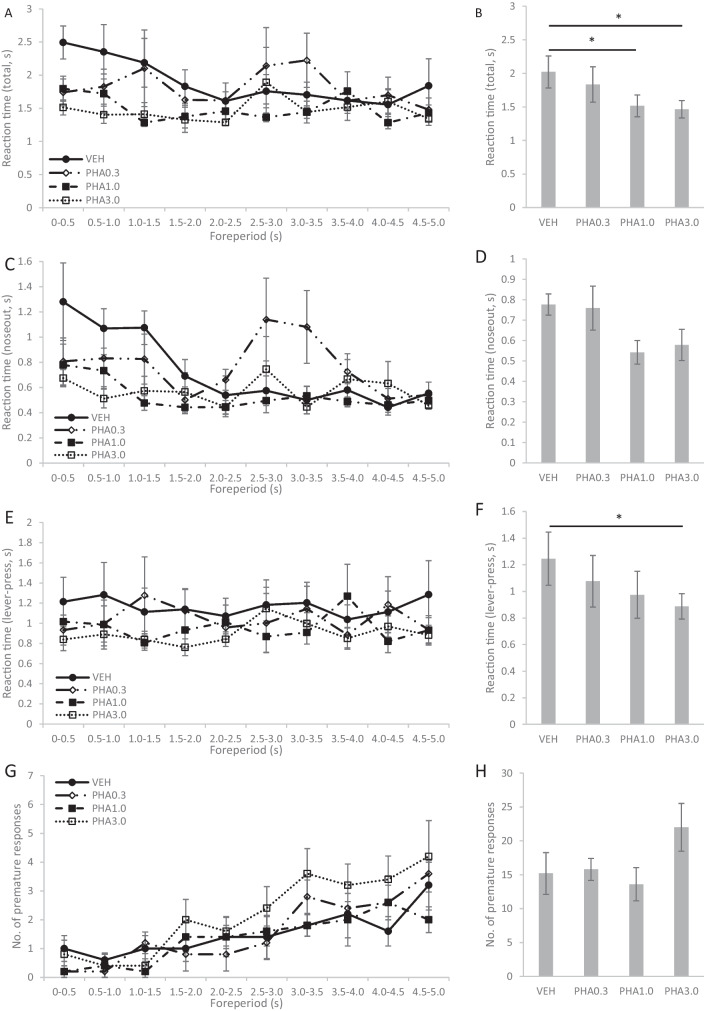
Effects of alpha7 nicotinic acetylcholine receptor agonist PHA-543613 on the performance of aged rats in the psychomotor vigilance task. **A**: Total reaction time (from the cue until pressing the lever) as a function of foreperiod length. **B**: Total reaction time averaged over all trials. **C**: Time elapsed from cue presentation until pulling the nose out of the feeder trough (noseout) as a function of foreperiod length. **D**: Noseout part of the reaction time averaged over all trials. **E**: Time elapsed from pulling the nose out of the feeder trough until pressing the lever (lever-pressing) as a function of foreperiod length. **F**: Lever-pressing part of the reaction time averaged over all trials. **G**: Number of premature responses as a function of foreperiod length. **H**: Total number of premature responses. Asterisk marks significant differences between treatments at the level of *p* < 0.05

Results of the second PVT experiment that investigated the effects of alpha7 nAChR agonist PHA-543613 on the cognitive performance of aged rats are summarized in Fig. 4. The experiment was conducted on 5 aged animals. The alpha7 nAChR agonist dose-dependently improved the reaction time of aged rats in the PVT (F(3, 12) = 2.877, *p* = 0.080) since 1.0 mg/kg and 3.0 mg/kg doses of PHA-543613 decreased the overall reaction time of aged rats compared to the treatment with vehicle (PHA1.0 vs. VEH: *p* = 0.041; PHA3.0 vs. VEH: *p* = 0.027). Interaction between the FOREPERIOD × TREATMENT factors showed that the effects of the treatments were dependent on the length of the foreperiod (F(27, 101.2) = 2.054; *p* = 0.005). Statistical data from the analysis of reaction time and its components in different ranges of the foreperiod length are summarized in Table 2. PHA-543613 improved the responsiveness of aged rats mainly for unexpected stimuli: reaction time in trials with 0–0.5 s foreperiod was significantly improved by all applied doses of PHA-543613, whereas reaction time in trials with 0.5–1.0 s foreperiod was only improved by the 1.0 mg/kg and 3.0 mg/kg doses of PHA-543613, while only a tendency was found for reaction time improvement after the treatment with 0.3 mg/kg dose of PHA-543613. Analysis of the decision phase of reaction time indicated similar interaction between effects (FOREPERIOD × TREATMENT: F(27, 101) = 2.192, *p* = 0.003), as “noseout” time was decreased by all doses of PHA-543613 in trials with 0–0.5 s foreperiod, and by 1.0 mg/kg and 3.0 mg/kg PHA-543613 in trials with 1.0–1.5 s foreperiod. PHA-543613 also showed a tendency to improve the overall speed of motor execution as the 3.0 mg/kg dose significantly decreased “lever-pressing” time (F(3, 12) = 2.813, *p* = 0.084; PHA3.0 vs. VEH: *p* = 0.017). The interaction between the foreperiod length and treatment with PHA-543613 in “lever-pressing” speed showed only a tendency to the significance level (FOREPERIOD × TREATMENT: F(27, 99) = 1.451, *p* = 0.095).

**Table 2:** Statistical data from the analysis of reaction time and its components in different ranges of the foreperiod length. If the main effect of the treatments were found significant (*p* < 0.05) or marginally significant (*p* < 0.1), then treatments with different doses of PHA-543613 were compared to vehicle treatment and uncorrected p-values were presented in the table

	Foreperiod									
	0–0.5 s	0.5–1.0 s	1.0–1.5 s	1.5–2.0 s	2.0–2.5 s	2.5–3.0 s	3.0–3.5 s	3.5–4.0 s	4.0–4.5 s	4.5–5.0 s
RT	F(3, 12) = 9.591 *p* = 0.002	F(3, 11) = 4.101 *p* = 0.035	F(3, 11) = 2.301 *p* = 0.132	F(3, 12) = 2.182 *p* = 0.143	F(3, 11) = 2.090 *p* = 0.160	F(3, 12) = 0.707 *p* = 0.567	F(3, 11) = 2.976 *p* = 0.077	F(3, 10) = 0.368 *p* = 0.778	F(3, 11) = 0.752 *p* = 0.544	F(3, 10) = 1.499 *p* = 0.275
PHA0.3	*p* = 0.002	*p* = 0.064					*p* = 0.163			
PHA1.0	*p* = 0.004	*p* = 0.034					*p* = 0.375			
PHA3.0	*p* < 0.001	*p* = 0.005					*p* = 0.375			
noseout	F(3, 12) = 4.454 *p* = 0.025	F(3, 12) = 2.208 *p* = 0.142	F(3, 12) = 3.758 *p* = 0.042	F(3, 12) = 0.825 *p* = 0.505	F(3, 12) = 1.346 *p* = 0.307	F(3, 11) = 1.823 *p* = 0.201	F(3, 12) = 3.561 *p* = 0.049	F(3, 11) = 0.629 *p* = 0.612	F(3, 11) = 0.417 *p* = 0.745	F(3, 11) = 0.338 *p* = 0.798
PHA0.3	*p* = 0.022		*p* = 0.229				*p* = 0.030			
PHA1.0	*p* = 0.017		*p* = 0.010				*p* = 0.903			
PHA3.0	*p* = 0.006		*p* = 0.025				*p* = 0.806			
press	F(3, 12) = 2.043 *p* = 0.162	F(3, 11) = 3.502 *p* = 0.053	F(3, 11) = 1.623 *p* = 0.239	F(3, 12) = 2.703 *p* = 0.092	F(3, 11) = 2.213 *p* = 0.144	F(3, 11) = 0.720 *p* = 0.561	F(3, 11) = 2.329 *p* = 0.130	F(3, 10) = 2.130 *p* = 0.159	F(3, 11) = 1.064 *p* = 0.405	F(3, 10) = 1.841 *p* = 0.207
PHA0.3		*p* = 0.032		*p* = 0.928						
PHA1.0		*p* = 0.030		*p* = 0.205						
PHA3.0		*p* = 0.011		*p* = 0.030						

**Fig. 5:**
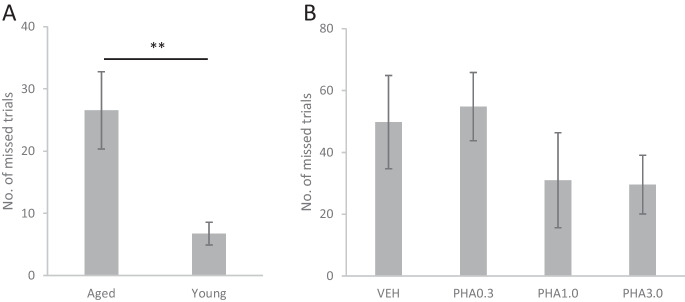
Number of missed trials in the psychomotor vigilance task experiments comparing the performance of aged and young rats (**A**), and effects of different doses of alpha7 nicotinic acetylcholine receptor agonist PHA-543613 on aged rats (**B**). Asterisks mark significant differences: ** *p* < 0.01

In summary, the treatment with PHA-543613 resulted in a global improvement of the reaction time (both in the decision and motor phases) of aged rats. However, PHA-543613 did not affect the number of premature responses (F(3, 12) = 1.462, *p* = 0.274) or the number of missed trials (F(3, 12) = 1.413, *p* = 0.287, Fig. 5B).

### Protein and mRNA expression levels of neuroimmune factors and alpha7 nAChRs in the brain of aged and young rats

**Fig. 6:**
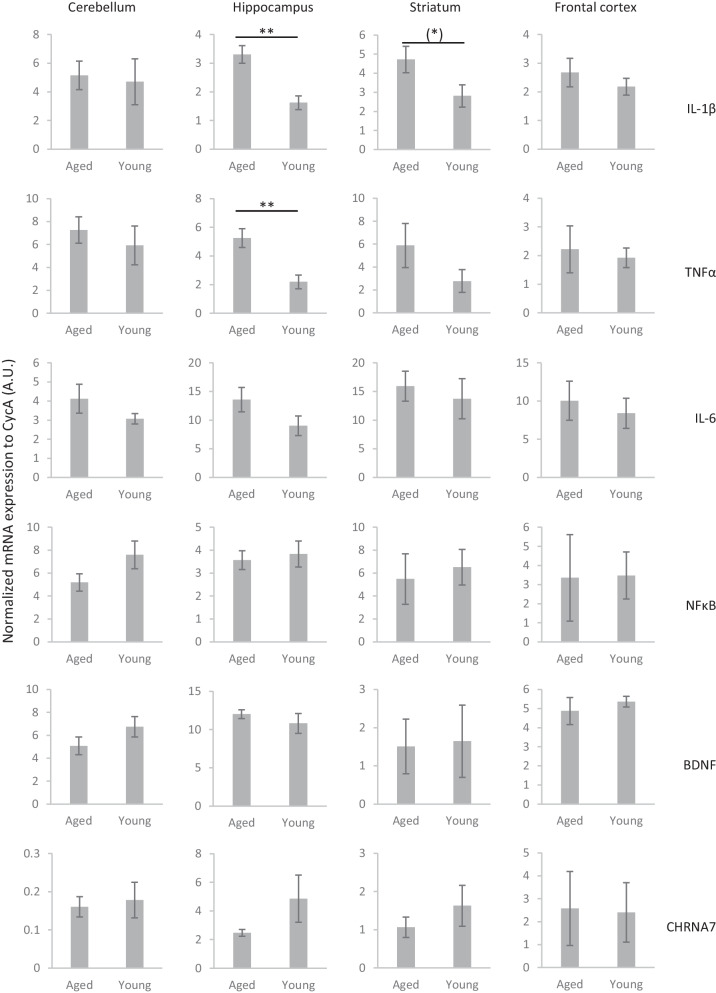
mRNA expression of cytokines, NFκB, BDNF, and alpha7 nicotinic acetylcholine receptor (CHRNA7) in different brain regions of aged and young rats measured with quantitative real-time PCR. Data are expressed in arbitrary units that indicate the mRNA expression of target genes normalized to CycA housekeeping gene expression. Asterisks mark significant differences between aged and young rats: (*) *p* < 0.1, ** *p* < 0.01

**Fig. 7:**
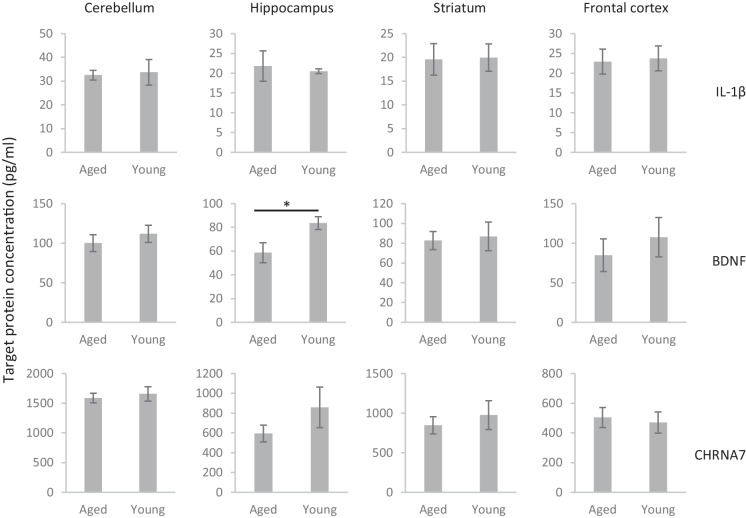
Expression of IL-1β, BDNF, and alpha7 nicotinic acetylcholine receptor (CHRNA7) proteins in different brain regions of aged and young rats measured with sandwich ELISA. Asterisk marks a significant difference between aged and young rats at the level of *p* < 0.05

Results of RT-PCR measurements indicated that the cognitive deficit of aged rats that was seen in both the MWM and the PVT was also accompanied by the increase in the occurrence of neuroinflammatory markers in the brain (Fig. 6). Namely, mRNA expression of major inflammatory factors IL-1β and TNFα were significantly upregulated in the hippocampus of aged rats compared to young adults (IL-1β: t(6) =  − 4.316, *p* = 0.005; TNFα: t(6) =  − 3.759, *p* = 0.009). However, there was no difference in the expression of NFκB and IL-6 in any of the examined brain regions. Furthermore, the level of BDNF protein in the hippocampus was lower in aged rats than in young rats (t(6) = 2.514, *p* = 0.046, Fig. 7). We did not find any differences in the brain expression of alpha7 nAChRs either in the mRNA or in the protein levels.

## Discussion

In summary, aged rats in the present study showed marked cognitive deficit in comparison with young adult rats both in the MWM task that tested spatial memory performance and in the PVT that tested vigilance and sustained attention of the rats. Aged rats reportedly underperform in other tasks measuring sustained attention like the 5-choice serial reaction time task [23], which was confirmed by our present results in the PVT. Furthermore, patients with Alzheimer’s disease or frontotemporal lobar degeneration also show impaired performance in the PVT, which is mainly manifested in the high number of omission errors or lapses (RT > 500 ms) [24]. In the rat PVT, omission errors are slightly differently defined than in the human PVT since the rats have to succeed in a fixation period which is usually not the case in the human PVT. Therefore, omission errors in human PVT may be considered an analog of the missed trials in the rat PVT. In the number of missed trials, aged rats performed badly similarly to aged humans. Thus, testing aged rats in the PVT may be a relevant preclinical model of cognitive deficits in age-related neurological and psychiatric diseases.

The cognitive impairment of aged rats was accompanied by increased mRNA expression of neuroinflammatory factors such as IL-1beta and TNFalfa and by a decrease in the protein expression of neurotrophic factor BDNF. Notably, a limitation of the present study is that the pharmacological experiment on aged rats ended only one day before the collection of brain samples. Thus, it is not known whether the treatment of PHA-543613 affected the mRNA and protein expression in their brains. Another limitation of the biochemical part of the study was that because of the small amount of brain samples and the need for a larger of samples for ELISA than RT-PCR, the protein products were not measured for all targeted genes. Furthermore, there were discrepancies between the measured mRNA and protein levels. The non-significant difference in the protein levels of IL-1beta may result from lower sensitivity of ELISA than RT-PCR measurements. However, our results that showed higher expression of inflammatory factors IL-1beta and TNFalpha are in line with earlier data that showed increased IL-1beta and TNFalpha levels in the brains of aged mice [25]. Interestingly, the increase of pro-inflammatory cytokines mainly occurred in the hippocampus during aging both in our present study and earlier studies [25]. Another study showed that chronic systemic inflammation in aged rats induces pro-inflammatory microglial phenotype in the hippocampus and also impairs hippocampal LTP which can be restored by inhibiting microglial activation with minocycline [26]. It can be concluded that there may be a direct link between the impairment of LTP generation in the hippocampus and the impaired cognitive performance in behavioral tasks as a result of neuroinflammation. Pro-inflammatory cytokines also show elevated levels in human patients with age-related neurocognitive disorders. In patients with amnestic mild cognitive impairment and Alzheimer’s disease, IL-1beta is expressed in higher levels in the plasma, cerebrospinal fluid (CSF), and in certain brain regions, especially in the frontal cortex and hippocampus, and the concentration of IL-1beta negatively correlates with cognitive performance [27–29]. Expression of TNFalpha was also shown to be increased in the plasma and CSF during aging, and its elevated plasma level is associated with decreased brain gray matter volume [30, 31].

Interestingly, the decrease in the expression of BDNF was only detected at the protein level, and there was no difference in the mRNA expression. However, such discrepancies between BDNF mRNA and protein measurements were also found in another study [32]. Earlier studies also failed to detect a decrease in the BDNF mRNA expression in aged animals [33, 34]. Such findings imply post-translational mechanisms that limit the availability of BDNF protein in certain brain areas of aged rats. Furthermore, the age-related changes in BDNF mRNA expression are also unequivocal in human studies: one study showed a negative correlation between BDNF expression and age, while another study showed that BDNF mRNA expression is not different between young adult and aged individuals [35, 36]. However, a consensus seems to be about the age-related changes in BDNF protein levels, as more studies showed a decrease in serum BDNF protein levels during aging [37, 38]. Interestingly, in the early phase of Alzheimer’s disease, a transient increase was found in the serum BDNF levels, which significantly decreases during the progression of the disease in correlation with the severity of dementia [39].

In our study, we treated aged rats with the alpha7 nAChR agonist PHA-543613. In the MWM, we did not find a significant difference between the performance of vehicle- and PHA-543613-treated aged rats. However, a non-significant increase was seen in the average time spent in the target quadrant in the probe trial, and aged rats treated with 3.0 mg/kg PHA-543613 did not underperform young rats. This result indicates a slight improvement in MWM performance after the PHA-543613 treatment. Our findings are in line with previous studies that investigated the cognitive enhancer effects of alpha7 nAChR agonist compounds [13]. In a series of experiments, we have earlier demonstrated the cognitive enhancer effects of PHA-543613 in different tasks using the scopolamine-induced transient amnesia model where PHA-543613 improved spatial working memory performance of scopolamine-treated rats in the T-maze spontaneous alternation task [40]. Furthermore, PHA-543613 also improved the long-term memory of scopolamine-treated rats in the MWM task [20]. We have also shown that the cognitive enhancer effects of PHA-543613 are synergistic with the similar effects of NMDA antagonist anti-Alzheimer drug memantine since combinations of subeffective doses of PHA-543613 and memantine also improved cognitive performance in both the spontaneous alternation task and the MWM [20, 41]. In the present study, PHA-543613 also improved the reaction time of aged rats to unexpected stimuli in the PVT. This is the first time to report the effects of an alpha7 nAChR agonist in the PVT. An earlier study using another task (5-choice continuous performance task, 5C-CPT) for the investigation of attention implied that nicotine-induced improvement in attention and vigilance can be accounted for the activity of alpha4beta2 nAChRs rather than alpha7 nAChRs [42]. However, a more recent study demonstrated in the 5C-CPT that alpha7 nAChR partial agonist encenicline improves attention and vigilance in low-attentive rats [43]. Another partial agonist of the alpha7 nAChR ABT-126 was shown to improve attention/vigilance scores in a randomized, double-blind, placebo-controlled trial in human schizophrenia patients [44]. Thus, the present results in the PVT confirm earlier data that showed improvement in attention and vigilance by treatment with alpha7 nAChR agonists. Our behavioral findings are also in line with earlier electrophysiological studies where alpha7 nAChR agonist PHA-543613 enhanced neuronal processing in the prefrontal cortex which is importantly involved in executive functions [45].

Targeting alpha7 nAChRs is also relevant in the aspect of neuroinflammation. Alpha7 nAChRs are involved in the cholinergic anti-inflammatory system (for a review see [46]). There is a growing body of evidence showing that activation of alpha7 nAChRs improves cognitive functions and decreases the levels of brain pro-inflammatory factors at the same time. It was shown that the anti-inflammatory actions of nicotine and cotinine are dependent on the alpha7 subtype of nAChRs [47, 48]. Similarly, the anti-inflammatory effects of vagus nerve stimulation also require the activation of alpha7 nAChRs [49]. Selective compounds acting at the alpha7 nAChR were also shown to induce a variety of effects that decrease neuroinflammation and promote neuroprotective processes in the brain and the periphery. Alpha7 nAChR agonist PNU282987 decreases the levels of IL-1beta and TNFalpha in a rat model of chronic migraine and also decreases the number of astrocytes and microglia in the hippocampus [50]. Mice with cognitive impairment due to intracerebroventricular streptozotocin injection were not only improved in memory tests by alpha7 nAChR agonist GTS-21 but the drug also blocked the streptozotocin-induced overproduction of TNFalpha, IL-1beta, and IL-6 in the hippocampus and the cortex [51]. Alpha7 nAChR agonists also decrease IL-1beta levels and increase BDNF levels in mice with perioperative neurocognitive disorder [52]. Positive allosteric modulators (PAM) of the alpha7 nAChR also effectively reduce neuroinflammation. JWX-A0108 improved the learning and memory function of Alzheimer’s disease model APP/PS1 mice by decreasing the expression of TNFalpha, IL-1beta, and IL-6 and also decreasing the phosphorylation of NF-kappaB [53]. Activation of alpha7 nAChRs also has direct effects on glial cells involved in inflammatory processes. PNU120596 decreased the expression of activation markers in microglia and astrocytes [54]. Furthermore, PNU120596 also has a neuroprotective effect by the attenuation of LPS-induced production of quinolic acid from microglia in the hippocampus and the prefrontal cortex [55]. On the other hand, alpha7 nAChR agonist PNU282987 induces the release of neuroprotective heme oxygenase-1 from microglial cells [56]. Although as a limitation of the present study we did not directly investigate the effects of PHA-543613 on neuroinflammatory markers, it is reasonable to consider that the observed beneficial pharmacological effects on cognitive performance may be – at least partly – accompanied by the amelioration of the progressive neuroinflammation of aged animals. Future studies should be designed to address the effects of PHA-543613 or other alpha7 nAChR agonists on neuroinflammatory markers in the brain.

In conclusion, our results suggest that beyond the previously described memory enhancer effects, the activation of alpha7 nAChRs may also be an effective strategy to improve attention and vigilance in age-related cognitive impairments. Targeting alpha7 nAChRs is especially relevant in age-related cognitive deficits. Future studies may shed more light on the causal connection between age-related attention deficit and neuroinflammation and may facilitate the development of novel drugs with combined effects on cognitive performance and neuroinflammation in age-related disorders of the brain and the mind.

### Supplementary Information

Below is the link to the electronic supplementary material.

Supplementary file1 (DOCX 429 KB)

## Data Availability

The authors declare that they make all experimental data available upon request to the corresponding author.
