# Supplementary material for: Increased brain cytokine level associated impairment of vigilance and memory in aged rats can be alleviated by alpha7 nicotinic acetylcholine receptor agonist treatment
Source: GeroScience. 2023 Nov 23;46(1):645–64. doi: 10.1007/s11357-023-01019-6 (PMC10828177; doi:10.1007/s11357-023-01019-6)
Supplement: Supplementary file 1 — Supplementary file1 (DOCX 429 KB) [file 11357_2023_1019_MOESM1_ESM.docx]

**Supplementary material**

**Methods**

**Training procedure for the psychomotor vigilance task (PVT)**

The steps of the PVT procedure are described in detail below. Progression from one step to the next was made if the rat performed 50 correct trials in two consecutive sessions.

Step 1: First, rats learn to nose-poke into the pellet delivery trough when the feeder light is on. The feeder light is continuously on until the rat performs a nosepoke, then a pellet is delivered as a reward (correct trial) and the feeder light is turned off for an intertrial interval (ITI) of 10 s.

Step 2: The feeder light is turned on for only 10 s, the animal has to perform a nosepoke during this interval. If a correct nose-poke is performed, the animal receives a pellet as a reward (correct trial), and a 25-second ITI starts with feeder lights turned off. If the animal does not perform a nosepoke, it is considered a missed trial, followed by a 30-second ITI with feeder lights turned off.

Step 3: The feeder light is turned on for 10 s, and the animal has to perform a nosepoke. The animal has to hold the nose in the pellet delivery trough for 500 ms until the feeder light turns off (correct trial). After a correct trial, the rat is rewarded with a pellet, and a 25-second ITI starts. If the animal does not perform a nose-poke, then it is considered a missed trial. If the animal performs a nosepoke but pulls the nose out of the pellet delivery trough earlier than the light had been turned off, then this is considered a premature trial. Both missed and premature trials are followed by the offset of the feeder light and an ITI of 30 s.

Step 4 is conducted similarly to Step 3, but the rat has to hold the nose in the pellet delivery trough for 1000 ms. The ITI is shorter, 20 s regardless of whether a correct or an erroneous trial was performed.

Step 5: In the 5th step, the animals have to learn to press the lever. The feeder light is turned on for 10 s and the animal has to perform a nosepoke. The animal has to hold the nose in the pellet delivery trough for 1000 ms until the feeder light turns off and the tricolor LED lights above the levers are turned on. Now, the rat immediately receives a reward pellet. Then, the rat has to press the lever to perform a correct trial. The animal has infinite time to perform the lever pressing. After pressing the lever, the rat is rewarded with an additional pellet, and a 20-second ITI starts. If the animal does not perform a nosepoke, then it is considered a missed trial. If the animal performs a nosepoke but pulls the nose out of the pellet delivery trough earlier than the light had been turned off, then this is considered a premature trial. Both missed and premature trials are followed by the offset of the feeder light and an ITI of 20 s.

Step 6a-d: The 6th step is split into four sub-steps. The task is the same as in the 5th step, but the foreperiod between the nosepoke and the cue for lever-pressing (feeder light off, and LED lights on) gradually increases as follows: 2000 ms in step 6a, 3000 ms in step 6b, 4000 ms in step 6c, and 5000 ms in step 6d.

Step 7: The task is the same as in step 6d (with a foreperiod of 5 s), but the rat does not receive a pellet after the nose-poke response, only after lever-pressing. However, the reward for lever-pressing is 2 pellets.

Step 8: The task is the same as in step 7, but the rat has only 10 s to press the lever after the onset of the cue (feeder light off and LED lights on). If the rat does not press the lever in the 10 s time window, it is considered an omission trial that is not rewarded and is followed by a 20 s ITI.

Step 9: The task is the same as in step 8, but the length of the foreperiod varies randomly between 0 and 5 s.

Step 10: The task is the same as in step 9, but the rat receives only one reward pellet after lever pressing. Step 10 is the protocol of the PVT, and rats that correctly perform it are considered trained for the PVT.

Troubleshooting: The most difficult step of the training procedure for rats was step 7, when there was no reward for nose-poking. For animals that could not complete step 7 for many sessions, we could help by decreasing the foreperiod length in step 7 to 3 s.

**Figures**


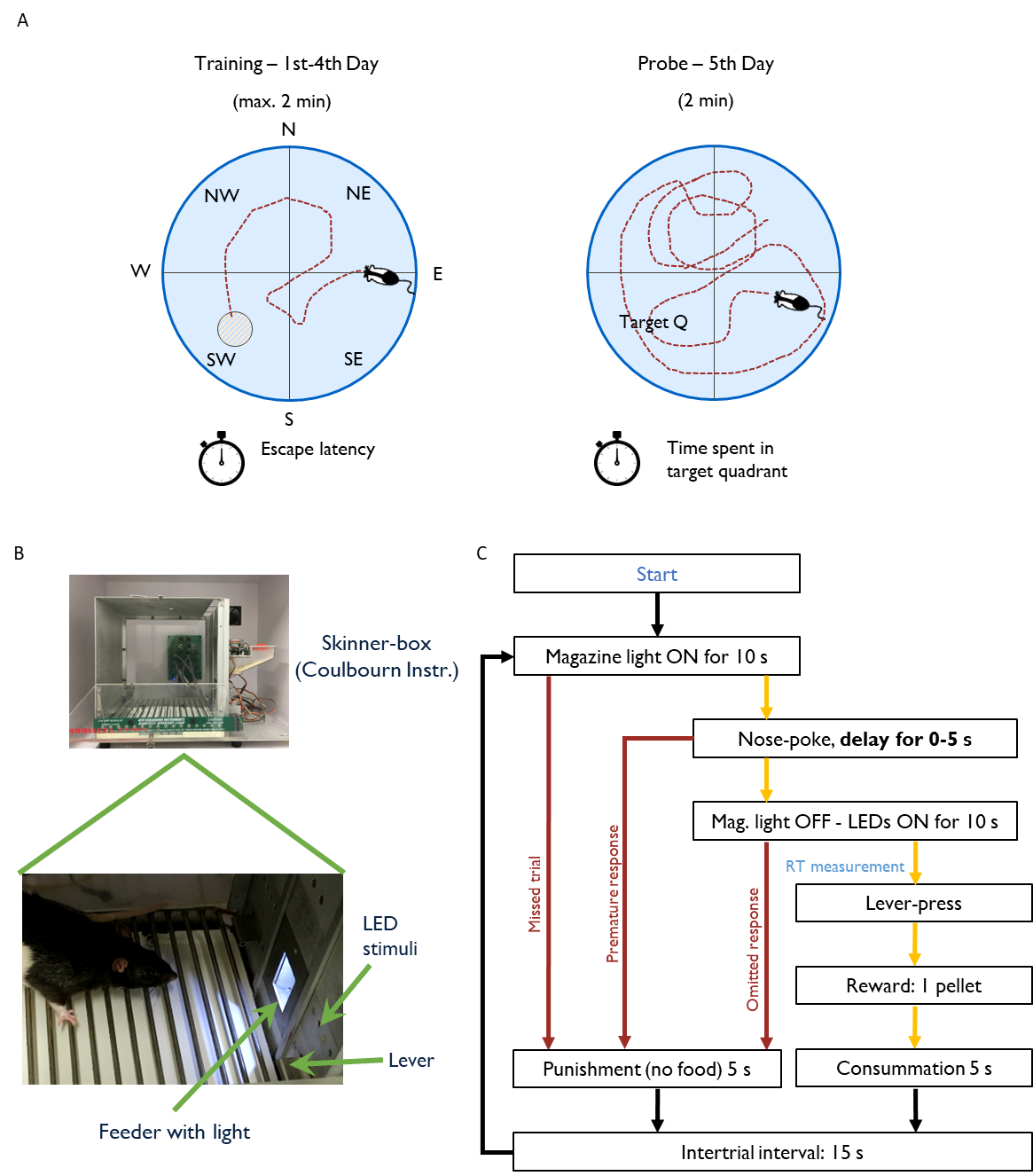


Fig. S1. Schematic presentation of the Morris Water Maze (A) and Psychomotor Vigilance Task (PVT, B-C). Panel B shows the operant conditioning apparatus that was used for the PVT. Panel C shows the protocol of the PVT.
